# Non-variceal Upper Gastrointestinal Bleeding and Its Endoscopic Management

**DOI:** 10.5152/tjg.2024.23507

**Published:** 2024-08-01

**Authors:** Reid D. Wasserman, William Abel, Klaus Monkemuller, Paul Yeaton, Vivek Kesar, Varun Kesar

**Affiliations:** 1 Department of Internal Medicine, Institute of Carilion Clinic, Riverside Circle, Roanoke, Virginia, United States; 2 Department of Gastroenterology, Institute of Carilion Clinic, Riverside Circle, Roanoke, Virginia, United States

**Keywords:** Upper gastrointestinal bleeding, endoscopic therapy, hemoclip, APC, peptic ulcer disease, thermal therapy

## Abstract

Upper gastrointestinal bleeding (UGIB) is a major cause of morbidity and mortality. Clinical symptoms that patients may present with include: hematemesis, coffee-ground emesis, melena, and hematochezia. Clinical signs can range from tachycardia to shock. The anatomical landmark that differentiates upper gastrointestinal (GI) bleeds from lower bleeds is the ligament of Treitz. The first steps of treating a patient who presents with signs of UGIB are resuscitation with appropriate fluids and blood products as necessary. The consideration of endoscopy and the urgency at which it should be performed is also vital during initial resuscitation. Endoscopic therapy should ideally be performed within 24 hours of presentation after initial stabilization with crystalloids and blood products. Intravenous proton pump inhibitors are the mainstay in the initial management of upper GI bleeding from a non-variceal etiology, and they should be administered in the acute setting to decrease the probability of high-risk stigmata seen during endoscopy. Pro-kinetic agents can be given 30 minutes to an hour before endoscopy and may aid in the diagnosis of UGIB. There are 3 broad categories of endoscopic management for UGIB: injection, thermal, and mechanical. Each endoscopic method can be used alone or in combination with others; however, the injection technique with epinephrine should always be used in conjunction with another method to increase the success of achieving hemostasis. In this review article, we will review the steps of triage and initial resuscitation in UGIB, causes of UGIB and their respective management, several endoscopic techniques and their effectiveness, and prognosis with a primary focus limited to non-variceal bleeding.

**Main Points**

- Upper gastrointestinal (GI) bleeding is one of the most frequent causes of emergency room visits that manifests with various presentations.
- Prompt resuscitation and endoscopy within 24 hours are the main management procedures for upper GI bleeding.
- Endoscopic management includes a multitude of therapeutic modalities including: injection, thermal, and mechanical to achieve hemostasis.

## Introduction

**Figure 1.**
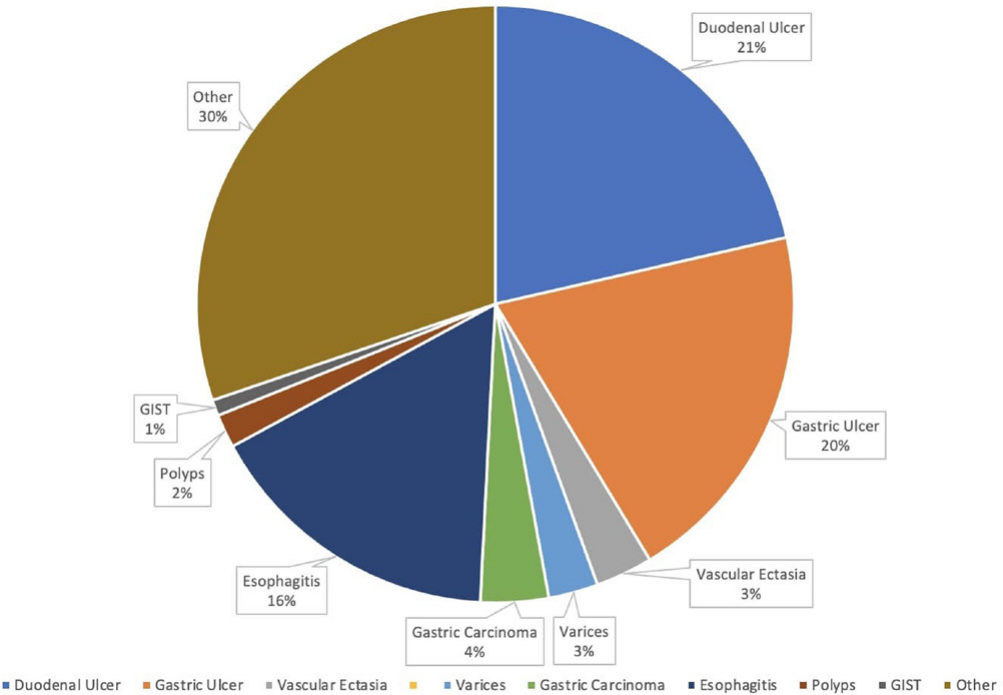
Etiologies of upper gastrointestinal bleeding by percentage.

**Figure 2.**
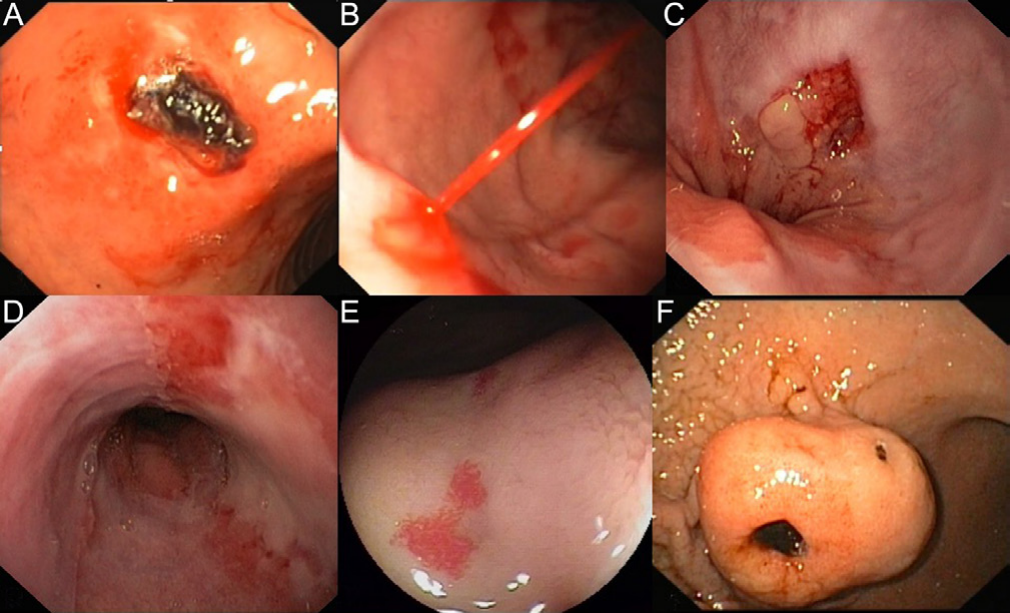
Various etiologies of upper gastrointestinal bleeding. A. Blood oozing peptic ulcer located in the duodenal bulb covered with an adherent clot. B. Bleeding duodenal Dieulafoy lesion on the greater curvature of the gastric body. C. Mallory–Weiss tear at typical location, on the distal esophagus, at the Z-line on the right side, which corresponds to the lesser curvature of the stomach. D. Severe erosive esophagitis, grade D based on the Los Angeles classification. E. Typical duodenal angiodysplasia. F. Large submucosal gastric tumor with ulcerated center. This is a gastrointestinal stromal tumor.

Upper gastrointestinal bleeding (UGIB) is one of the most frequent causes of emergency room visits and hospital admissions. Every year, patients presenting with UGIB account for 80-150 per 100 000 population, with estimated mortality rates between 2% and 15%.^1^ Common presentations include: coffee-ground emesis, vomiting bright red blood (hematemesis), bright red blood per rectum (hematochezia), or black, tarry stools (melena). Symptoms and complaints of patients may not directly include the aforementioned, but rather veiled by symptoms such as: syncopal episodes, shortness of breath, chest pain, fatigue, and weakness. Upper gastrointestinal bleeding may be acute, requiring intervention within 24 hours, occult (presenting without apparent visual blood loss), or chronic (over months to years). Peptic ulcer disease (PUD) is one of the major causes of UGIB, which makes up about 40%-50% of cases (Figure 1).^1-3^ Other common etiologies include: esophagitis, Mallory–Weiss tear, esophageal varices, gastritis, Dieulafoy lesions, arteriovenous malformations, and malignancy (Figure 2). Rare causes of UGIB include: aortoenteric fistula, hemobilia, and hemosuccus pancreaticus. Common risk factors for UGIB include: prior UGIB, alcohol use, frequent and extensive use of nonsteroidal anti-inflammatory drugs, older age, liver disease, and antiplatelet/anticoagulation therapy such as aspirin, clopidogrel, apixaban, and warfarin.^4^

Endoscopic management of UGIB is the mainstay of treatment. Endoscopy should be performed within 24 hours for patients who are hemodynamically unstable (hypotensive and tachycardic). Patients who present with hematemesis and a history of cirrhosis or a documented history of esophageal varices/bleeding should have endoscopy performed within 12 hours.^2^ Initial hemodynamic stabilization is imperative for prognosis. This is achieved with prompt intravascular volume replacement and transfusion with packed red blood cells (pRBC) to a goal of 7 g/dL, and 8 g/dL in patients with a significant cardiac history.^5^ There are 3 broad categories of therapeutic endoscopy to achieve hemostasis including injection, thermal, and mechanical.

### Initial Triage

Initial triage of patients includes measuring and obtaining: vital signs, history of present illness, and physical exam, including digital rectal exam. These first steps are crucial to assess and evaluate if prompt endoscopic intervention is necessary, or if delayed intervention/discharge with outpatient management is appropriate. However, hemodynamic stability, vital signs, and overall presentation do not predict severity or outcome of UGIB.^6,7^ Risk stratification tools can be utilized to classify low and high-risk patients that help determine clinical decisions. Three common risk scores are: Glascow Blatchford Score (GBS), Rockall Score, and AIMS65 score. The GBS takes into account the patient’s history and vital signs to produce a stratified score. A GBS > 0 was 99%-100% sensitive and up to 44% specific for identifying a severe bleed in 5 studies.^7-11^ Patients who score a 0-1 on this tool are at lower risk, and discharge with outpatient follow-up can be considered in this circumstance. The Rockall Score is based on the patient’s age, shock status, and comorbidities. In 3 studies, the GBS outperformed the Rockall Score with regards to predicting patients at high risk for intervention.^7-9^ AIMS65 is an aggregate score of 5 pre-endoscopy variables that combine factors of liver function (albumin, INR) with patient presentation (presence of encephalopathy), vital signs, and age. Studies that have compared the AIMS65 against the GBS and Rockall Score have shown the AIMS65 score to be more predictive of mortality and the need for intensive care unit.^12^

After triage and risk stratification, initial resuscitation with crystalloids should be employed to achieve hemodynamic stability (with supplying intravenous (IV) fluids and blood products as necessary). Prompt IV access with large bore catheters (14 gauge or 16 gauge) is imperative for adequate resuscitation in the hemodynamically unstable patient. Patients with a large loss of blood volume or those with hemoglobin less than 7 g/dL (except in patients with pre-existing coronary artery disease) should be transfused with pRBCs. Additionally, necessary blood products should be administered if the platelet count is less than 50 000/mm^3^ or evidence of coagulopathy (prothrombin time greater than 15 seconds) is present.^13^ Generous administration of pRBCs to compensate for a large volume of blood loss is not recommended, and studies have shown that providing blood at a certain threshold with limitation significantly improved outcomes.^13^

The administration of IV proton pump inhibitors (PPI) is standard practice when patients are presenting with signs of UGIB. However, evidence suggests PPIs do not affect mortality or outcome of UGIB.^14,15^ A Cochrane meta-analysis showed that PPIs decrease the probability of visualizing ulcers with high-risk stigmata during endoscopy.^6,14,15^ As such, the 2021 American Board of Gastroenterology guidelines do not recommend for nor against pre-endoscopic PPI therapy.^14^ However, based on the authors’ primary experience, PPI therapy during active bleeding is beneficial, specifically if prompt endoscopic services are not available. Intravenous PPI may be given as an intermittent twice daily dose and then either continued or transitioned to oral based on endoscopic findings.

The use of a nasogastric tube (NGT) may be used to help identify UGIB in selective patients; however, it cannot be used to definitively diagnose UGIB. If bright red blood is apparent during gastric suctioning, this may be a sign of high-risk lesions. If no red blood is apparent, or coffee-ground material is aspirated, NGT aspiration is less useful to determine the presence of high-risk stigmata or UGIB. Additionally, inability to visualize bright red blood with gastric aspiration is not highly sensitive for ruling out UGIB, as up to 15% of patients with active bleeding do not manifest consistent findings with nasogastric lavage.^5,6^

Visualization of anatomical structures is of utmost importance to distinguish abnormal features or pathology during endoscopy. Erythromycin and metoclopramide are 2 medications that can be administered prior to endoscopy. The agents increase gastric motility and may be used to aid the endoscopist in identifying the site of interest. A meta-analysis that compared the efficacy of the 2 agents revealed that administration up to 2 hours prior to endoscopy led to increased visualization that allowed endoscopists to perform adequate intervention, thus negating the need for repeat endoscopy.^5^ The endoscopic management of UGIB is time-sensitive, and studies have shown specific outcomes regarding the timing of intervention in the acute setting. A randomized control trial (RCT) in 2020 showed no difference in mortality or decrease in recurrent bleeding when patients underwent endoscopic intervention within 6 hours vs. within 24 hours of the initial evalutation.^16^ Retrospective analysis has shown that endoscopy performed within 6-8 hours of patient presentation resulted in revealing higher-risk lesions, although the outcome was not influenced.^17-19^ Timing and urgency of upper endoscopy also rely on clinical presentation and patients’ comorbidities. The American College of Gastroenterology guidelines support the aforementioned literature of performing endoscopic intervention within 24 hours for patients presenting with non-variceal UGIB who are hemodynamically unstable.^14,16^

## Types of Endoscopic Therapies

**Figure 3.**
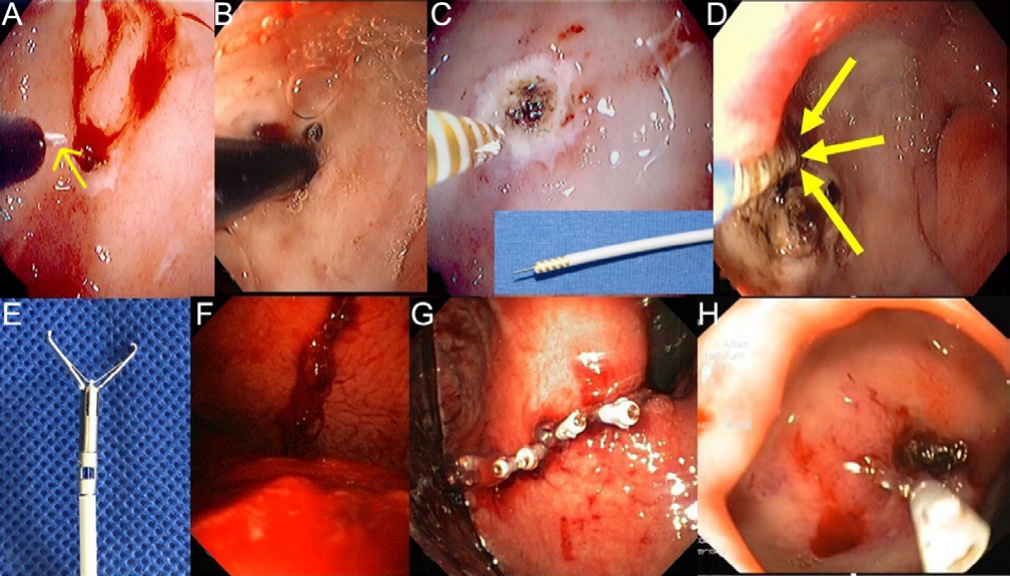
Common accessories employed for endoscopic therapy. (A) Injection needle (yellow arrow). (B) Epinephrine-saline mix 1 : 20 000 injected into the bleeding ulcer. (C) Bipolar electrocoagulation using the gold-probe (also see insert). The gold probe also has a needle, facilitating a dual endoscopic therapy. (D) The arrows show the gold probe tip, tightly applied against the visible vessel. This maneuver is essential to induce adequate cauterization and hemostasis. (E) Hemoclip. (F) Large bleeding lesion in the stomach in a patient who underwent surgical enucleation of a leiomyoma. (G) Technique of “zipper-clipping” resulting in adequate hemostasis. (H) Hemoclip placed on a visible vessel with an adherent clot.

There are a multitude of endoscopic therapies to treat UGIB which includes: injection, thermal, mechanical, and hemostatic powders. (Table 1, Figure 3). Injection therapy of diluted epinephrine (1 : 20 000) is the oldest method for obtaining endoscopic hemostasis,^17^ and a meta-analysis of 4 RCTs showed it is the least effective compared to other monotherapies such as clips and bipolar electrocoagulation.^14^ Additionally, epinephrine combination therapy is more effective than monotherapy,^14^ and should be used as dual therapy to increase the probability of achieving hemostasis. The injection of epinephrine compresses the surrounding tissue to tamponade the bleeding site. Polidocanol is another injectable agent that has been studied and has been effective up to 90% as shown in one study.^20^ However, this material does come with complications such as mucosal necrosis and risk of perforation.^20^ Ethanol is another injectable substance that has been used to treat upper GI bleeding. One prospective study showed successful hemostasis in 29 out of 33 patients with ethanol injection for peptic ulcer bleeding that required second look endoscopy.^21^

Mechanical devices for endoscopic treatment include endoscopic clips and metallic devices that achieve hemostasis by approximating tissue with tamponade of the bleeding site or compression of the bleeding vessel. There are 2 broad categories of clips: through-the-scope clips (TTSC) and cap-mounted (over-the-scope (OTSC)) clips. Studies have compared endoscopic clips to dual therapy with epinephrine injection and bipolar thermal (BPT) cautery, with comparable rates of achieving hemostasis.^17,22^ A meta-analysis showed that endoscopic clips were superior at achieving hemostasis when compared with injection therapy alone (87% vs. 75% respectively).^17,22^ Additionally, a meta-analysis showed that using mechanical clips or thermal therapy as a second treatment modality after epinephrine injection has been shown to decrease the risk of rebleeding; however, mechanical clips did not show a significant reduction when compared to thermal therapy.^23^ An RCT that compared endoscopic clips with hypertonic saline–epinephrine injection for treatment of peptic ulcers showed a significant advantage with regards to safety and efficacy with clips, and the combination of the two did not provide a substantial advantage.^24^ The endoscopist may choose to use hemoclips over thermal therapy for different etiologies in UGIB including Mallory–Weiss tears (which would allow for approximation of tissue) or the presence of coagulopathy.

Through-the-scope clips are typically the first-line therapy for ulcer-related UGIB;^25^ however, limitations of TTSC include difficult anatomic locations and large (>2 cm) or fibrotic ulcers. Cap-mounted clips were originally developed to close large surface area mucosal defects, but they have also been utilized for the treatment of UGIB. They are larger-caliber clips (typically 11, 12, and 14 mm), which can grasp a larger and deeper surface area of mucosa with higher pressure, which may improve hemostasis.^25^ Two proprietary devices available for endoscopy are Ovesco, which is an OTSC system, and the padlock system. The OTSC system is primarily used in patients who have had an initial therapeutic endoscopy without success in achieving hemostasis. A prospective study, which included 66 patients that compared traditional endoscopic clips with OTSC revealed a statistically significant difference in the ability to achieve hemostasis and prevent rebleeding from peptic ulcers in the OTSC group (16.1% vs. 91% respectively).^26^ Additionally, the STING-2 trial was a prospective RCT of 100 patients that compared first-line OTSC with standard endoclips in high-risk patients, which resulted in a clinical success rate of 91.7%.^27^ Recent meta-analysis of 10 studies, including 4 RCTs, showed OTSCs had an overall lower risk of 7-day and 30-day rebleeding and decreased procedure time when compared to standard therapy.^14,16,17^ In some circumstances, localizing a bleeding lesion and manipulating anatomy may be difficult with a standard endoscope. The use of caps has been described to aid in achieving hemostasis with hemoclip placement. A small prospective study that included 10 patients with sphincterotomy-associated bleeding showed a 90% success rate.^28^ The cap allows the endoscopist to navigate mucosal folds that may hinder an adequate view of the lesion and also improves stability to aid with localization and therapeutic management.^28^

Thermal therapy is an additional endoscopic modality that is used to achieve hemostasis via heat or cold. Heat induces hemostasis by producing inflammation at the directed site, as well as causing nearby blood vessel vasoconstriction and potentiation of coagulation factors.^29^ There are 2 broad categories of thermal therapy: direct contact and noncontact devices. Of the contact devices, BPT cautery is frequently used among endoscopists.^17^ A meta-analysis showed that direct thermal contact heating probe and bipolar coagulation reduce bleeding and mortality compared with no endoscopy.^14,30^ The BPT is particularly helpful when the ulcer has a fibrotic base, making it difficult to have tissue apposition. Bipolar probes, similar to heater probes, are applied directly to the source of bleeding with light pressure while administering 4-6 pulses of coagulation, each lasting up to 12 seconds.^17^ Additionally, a prospective study showed that patients presenting with UGIB from peptic ulcers who underwent dual therapy of epinephrine injection and bipolar coagulation provided an advantage in decreasing the risk of rebleeding and decreasing the requirement for blood transfusion.^31^ Heater probes are another contact thermal device that is frequently used, but care must be taken as coagulation is achieved by placing pressure directly over the source of bleeding. Perforation is a possible complication of heater probe coagulation because direct pressure from the probe can cause deeper coagulation affecting tissue beyond the mucosa. Discrete pulses with light pressure can be applied to prevent this complication.^32^

Hemostatic coagulation graspers use force and coagulation to cauterize blood vessels. Hemostatic forceps are often used during endoscopic submucosal dissection and have been used for bleeding from peptic ulcers as well. Hemostatic forceps are useful in that the endoscopist may directly grasp a visible vessel, or use the tip of the forceps to apply cautery to achieve hemostasis. A RCT of monopolar hemostatic forceps compared with hemoclips showed a significantly higher initial hemostasis rate (98.2 vs. 80.4% respectively), and the risk of rebleeding over 7 days was decreased compared to the standard hemoclip group.^33^ Cap-assisted thermal treatment may aid with the management of UGIB, as previously shown with hemoclips. Mucosal folds and altered anatomy may make standard use of hemostatic forceps more difficult. Caps allow for better stabilization, friction, and increased diameter of the scope for easier maneuverability.^28^

Argon plasma coagulation (APC) is an endoscopic intervention that utilizes thermal conduction via argon gas that is emitted from a monopolar electrode. The probe is brought to a close proximity (2-8 mm) of the bleeding lesion without contacting it, and argon gas is ionized resulting in conduction of current and coagulation of nearby tissue, thereby achieving hemostasis.^34,35^ A meta-analysis that compared APC among other interventional modalities showed no significant difference in outcomes.^14,25,34^ One prospective study of APC to treat high-risk PUD bleeding showed that APC was safe and effective and comparable to the outcomes of the heater probe.^36^ A RCT that compared epinephrine injection with the heat probe with injection and APC showed a similar rate of primary hemostasis (95.9% vs. 97.7%).^37^ Similarly, a prospective RCT that compared hemostatic forceps to APC for the treatment of high-risk PUD showed noninferiority with a 96% success rate.^38^ Argon plasma coagulation is also useful particularly in superficial lesions and etiologies of UGIB such as gastric antral vascular ectasia (GAVE), arterio-venous malformations, and tumor bleeding.^29^ A retrospective study of the efficacy of APC for tumor bleeding showed a 100% success rate in initial hemostasis.^39^

Another method of achieving temporary hemostasis in UGIB is hemostatic powders that are diffusely dispersed over the site of bleeding during endoscopy. There are 3 commercial hemostatic sprays available: Hemospray (TC-325), Ankaferd blood stopper, and EndoClot, but only Hemospray (TC-325) and EndoClot are Food and Drug Administration approved in the United States.^29^ Hemospray is a hemostatic powder that is topically spread over a bleeding lesion. It achieves temporary hemostasis by activating platelet aggregation that ultimately leads to activation of coagulation factors. The delivery catheter is brought within close proximity to the site of bleeding and administered until the affected area has a thin layer of the agent that potentiates hemostasis. One large RCT that compared the use of Hemospray and standard endoscopic therapy for non-variceal UGIB showed less further bleeding at 30 days with Hemospray.^14,40^ Additionally, a large meta-analysis of 19 studies showed that Hemospray was successful in achieving initial hemostasis with a success rate of 92%; however, the study revealed a 20% early rebleeding rate.^41^ Large prospective studies are lacking to conclude adequate efficacy for Ankaferd Blood Stopper, but a retrospective case series showed hemostasis was achievable in 26 patients.^42^ Kurt et al^43^ has shown immediate hemostasis in 10 patients with bleeding from GI tumors. One prospective study that evaluated EndoClot showed the ability to stop UGIB in 64% of patients.^44^ Another observational study of 21 patients that reviewed the effectiveness of EndoClot showed a 100% rate of initial hemostasis.^45^ Hemospray is not typically used as a primary method of achieving hemostasis but is often used for temporary hemostasis in an unstable patient that likely requires more aggressive intervention. The primary authors believe Hemospray is helpful for multiple areas of bleeding, as one may see in the setting of tumor bleeding.

Endoscopic doppler probe is not used as a therapeutic intervention during UGIB, but rather can be used as an adjunct diagnostic modality to assess local blood supply, efficacy of treatment, and stratify those at higher risk of repeat bleeding. The probe is passed into the endoscopic instrument channel, and gentle contact is made around the surrounding bleeding site to determine the location of blood vessels that may be amenable to endoscopic intervention. Pulsatile doppler signals can indicate the presence and path of bleeding vessels. After endoscopic hemostasis is achieved, the probe can be placed around the site to determine if vessels were appropriately coagulated. The absence of a doppler signal is consistent with successful hemostasis of a bleeding lesion. Lesions that have a persistent dopplerable signal are considered higher risk for rebleeding.^29^

## Types of Lesions and Endoscopic Management

Peptic ulcer disease accounts for 30%-60% of UGIB.^6^ Non-steroidal anti-inflammatory drugs and *Helicobacter pylori* (Hp) are the 2 major causes of PUD. *Helicobacter pylori* infection is common among the population in developing countries with some estimates as high as 80%.^34^ The risk of acquiring PUD from Hp varies among developed countries, but has been reported from 3% in the United States to 25% in Japan.^34^ The Forrest Classification is a common tool used during endoscopy to predict the risk of rebleeding and mortality of peptic ulcers.^2^ The Forrest classification stratifies ulcers based on their appearance during endoscopy.^46^ Endoscopic treatment for PUD entails injection, mechanical, and thermal modalities. If the endoscopist chooses to inject epinephrine, this modality should not be used as monotherapy. An additional therapeutic modality such as cauterization with monopolar or bipolar probe or endoscopic clips increases the efficacy of hemostasis compared with epinephrine alone in reducing rebleeding risk and potential surgery.^46^ Mechanical therapy with endoscopic clips has shown to be effective in achieving initial hemostasis for high-risk peptic ulcers up to 95%, as shown in a prospective study that treated 40 patients with PUD with high-risk stigmata.^47^ Additionally, a large meta-analysis that included 28 RCTs and reviewed the treatment of PUD showed that endoscopic clips were more effective than injection therapy alone in preventing rebleeding (with a number needed to treat of 7), but no difference with regards to initial hemostasis.^48^ This meta-analysis also revealed no statistical differences in outcomes between thermal coagulation and hemoclips with regards to initial hemostasis, rebleeding, and mortality.^48^ As previously mentioned, dual therapy of epinephrine injection and bipolar coagulation was shown to be effective in peptic ulcer bleeding. An RCT compared 58 patients who were treated with both injection and BPT compared with 56 patients in the control group that were only treated with BPT. Results showed combination therapy was superior in achieving initial hemostasis with an absolute risk reduction of 31.6%.^36^

Endoscopic management of tumor bleeding is not as effective at achieving hemostasis compared with other causes of UGIB due to the propensity and unpredictable nature for rebleeding along with poor wound healing. Contact thermal therapy is an effective option for initial hemostasis as shown in a retrospective analysis, which compared the efficacy and success of heater probe and bipolar electrocautery in seven patients. All of the patients had successful initial hemostasis, but the study also revealed a similar 30-day rebleeding rate (33%).^49^ The authors concluded that although endoscopic therapy may be effective initially, there appears to be high mortality, rebleeding risk, and requirement of surgery associated with tumor bleeding that is not dependent on the type of therapy.^49,50^ Argon plasma coagulation and Hemospray are other modalities that are used in GI-related tumor bleeding. Upon review of the literature, studies regarding endoscopic intervention for UGIB due to malignancy are limited, and those that have been conducted include a small cohort of patients. This may be limited to the high-risk of rebleeding due to the nature of these malignancies.

Mallory–Weiss tear is defined by a linear, mucosal laceration of the esophagus that is usually caused by retching or vomiting. This condition is commonly seen in alcoholics and bullemics. It is common for tears to heal without endoscopic intervention, as bleeding usually stops spontaneously; however, if bleeding persists, diagnostic endoscopy would need to be performed.^46^ Several endoscopic methods can be employed for treating Mallory–Weiss tear including: mechanical, injection, and contact thermal treatment. Injection therapy with epinephrine is useful to achieve hemostasis as shown in a RCT of 63 patients. Two patients had rebleeding in the study group when compared with 8 in the control group (6.2% vs. 25.8% respectively).^51^ However, injection and another therapeutic modality should be utilized concurrently since there is a higher risk of rebleeding if single therapy with epinephrine is used.^52^ Hemostatic clips are another effective option to achieve initial hemostasis and prevent rebleeding as seen in a retrospective study of 47 patients (with an initial hemostasis success rate of 100%).^53^ Endoscopic band ligation (EBL) has also been studied for treatment of Mallory–Weiss tears. One study that compared EBL with clips and epinephrine injection combined showed that rebleeding was higher in the clip and injection group when compared with EBL (18% vs. 0%, respectively).^54^ Cauterization with monopolar or bipolar therapy is another option, however, it is less effective due to dissipation of heat while manipulating the probe in bloody or salivary secretions, rendering the field “wet.”^52^ One study showed that 10 out of 13 patients were successfully treated with electrocoagulation.^55^

A Dieulafoy lesion is a large, aberrant, submucosal artery that is known for intermittent bleeding. The most common site is the stomach, as it has been described to present frequently along the lesser curvature; up to 95% of lesions are found within close proximity to the gastroesophageal junction.^56^ Clinical presentation can vary, but commonly presents with onset of hematemesis, melena, or hematochezia.^57^ It is usually diagnosed via endoscopy, however, multiple endoscopies may be required for diagnosis due to the intermittent nature of the lesion, size, and location. Additionally, index endoscopy may not capture the abnormal artery in an acute state of bleeding. Endoscopic ultrasound to help identify the aberrant artery has been reported in the literature. Endoscopic management that may be used for Dieulafoy lesions include: injection, thermal, and mechanical. A study that compared mechanical clips and injection showed that clips were superior to injection with regards to initial hemostasis and rebleeding (91.7% vs. 75%, respectively).^58^ Injection with epinephrine should not be used alone due to the possibility of rebleeding.^57^ Epinephrine injection can be used before direct therapeutic intervention as a tamponade device to reduce the potential for excessive bleeding. Similarly to Mallory–Weiss tear, direct contact in a wet field with heater probe or bipolar coagulation are often not adequate since Dieulafoy lesions are typically covered in blood, and heat is dissipated resulting in ineffective hemostasis. Argon plasma coagulation can be a useful method to treat these lesions as shown in one study that reviewed 23 lesions with a 100% success rate of initial hemostasis.^59^ If endoscopy fails to localize the lesion, angiography can be a useful tool for identification.

**Figure 4.**
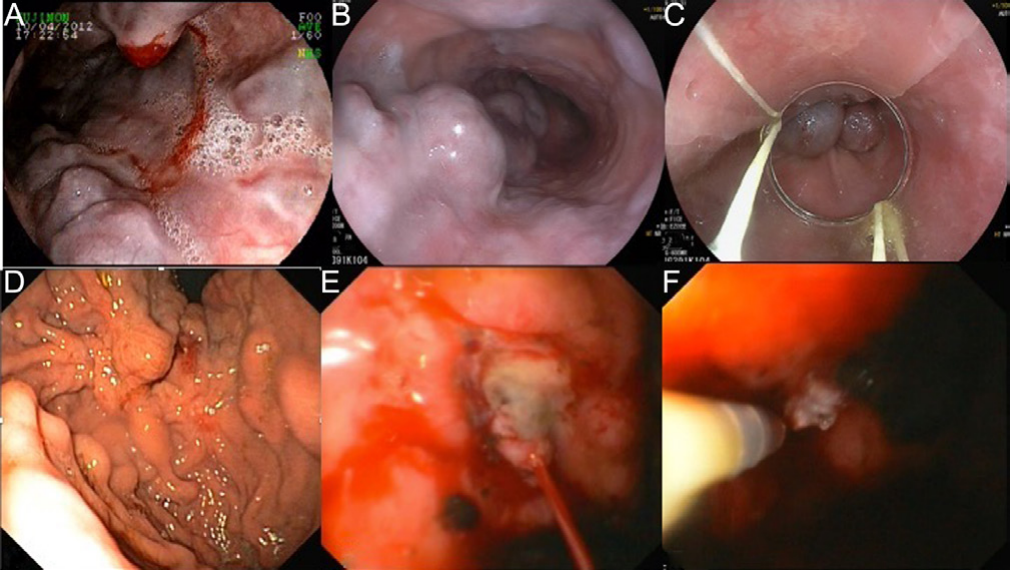
Esophagogastric varices. (A). Bleeding distal esophageal varices. Notice the blood emanating from the “nipple sign.” (B) Grade 4 esophageal varices based on the Paquet classification. (C) The esophageal varices were banded with excellent proximal decompression. (D) Gastric varices extending from the gastroesophageal junction to the fundus, which corresponds to type Gastroesophageal varix (GOV) II based on the Sarin classification. (E) Bleeding gastric varix. (F) Successful hemostasis of bleeding gastric varix using glue (Histoacryl) injection.

**Figure 5.**
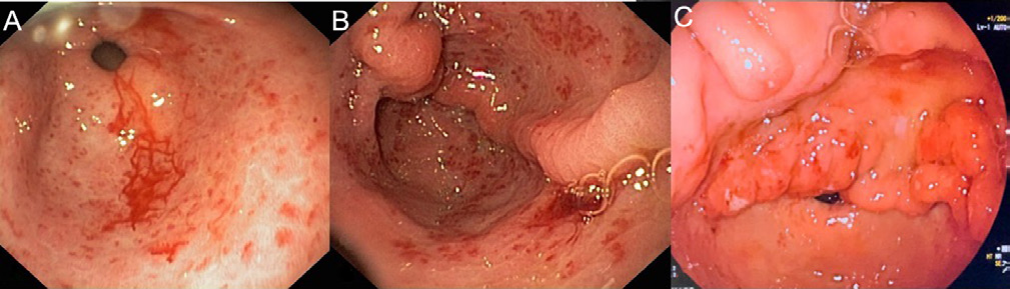
Gastric antral vascular ectasia (GAVE). (A) Honeycomb type with active bleeding. (B) We call this the large fold type. Usually, there are no folds in the antrum. (C) Typical appearance of nodular type GAVE.

Gastric antral vascular ectasia (also known as “watermelon stomach”) is more likely to cause chronic anemia over time, but is possible to present with acute drops in hemoglobin as well. Endoscopic appearance reveals dilated blood vessels in the antrum that form a striped pattern that spread to the pylorus of the stomach (Figure 4). There are several postulations of the causes of GAVE including chronic liver disease and autoimmune disease. Gastric antral vascular ectasia is not as prevalent as other etiologies of UGIB, however, it consists of 4% of non-variceal upper GI bleeding.^60^ Patients may not present with any symptoms and may be undiagnosed until upper endoscopy is performed (Figure 5). Patients may also present with hematemesis or melena. Endoscopic intervention can be used to treat GAVE, and the most common modalities include: cryotherapy, APC, radiofrequency ablation, and EBL; however, of the 3 aforementioned, APC is the most common to treat GAVE. Large studies comparing the aforementioned treatments are currently lacking, but APC appears to have the highest reported efficacy, ranging from 90% to 100%.^61^ A meta-analysis that reviewed EBL to treat GAVE showed a pooled treatment rate of 81%.^62^ Studies that have compared EBL with APC have been performed and actually showed a lower rebleeding rate with EBL (8% vs. 68%, respectively).^63^ Once used as a method to treat refractory bleeding for GAVE, EBL may be used as an alternative treatment given its high efficacy rate (as high as100% in another study) in achieving hemostasis.^64^

This review article explores relevant retrospective and prospective studies and clinical trials for evidence. Studies that have shown a clear decrease in morbidity/mortality with regards to the management of upper GI bleeding are included. Limitations of this review are lacking the inclusion of the lesser common etiologies of upper GI bleeding and their management. Last, when RCTs were not available to support the use of specific endoscopic modalities due to limited data, smaller retrospective and prospective studies are used for substantiation.

## Conclusion

Upper GI bleeding is one of the most frequent conditions encountered in the emergency department. When patients present with symptoms of UGIB, which include: melena, hematemesis, coffee-ground emesis, or hematochezia, prompt hemodynamic resuscitation is the first step in management. Prompt endoscopic intervention should be considered within 24 hours, or sooner if the patient has persistent signs of hemorrhage and hemodynamic instability. Peptic ulcer disease is the most common etiology of upper GI bleeds. There are 3 broad categories of endoscopic management for UGIB: injection, thermal, and mechanical. Each endoscopic method can be used alone or in combination with others; however, the injection technique with epinephrine alone is not as effective as dual therapy with an additional endoscopic modality, as this increases the success of achieving hemostasis. Further research should include a comparison of the three broad categories of endoscopic management and their efficacy in different etiologies of upper GI bleeding.

## Figures and Tables

**Table 1.** Endoscopic Therapies to Treat Upper Gastrointestinal Bleeding

**Injection**	**Mechanical**	**Thermal**	**Hemostatic Powder**
Epinephrine (1 : 10 000/20 000)	Through-the-scope clips: Endoscopic hemoclips Cap-mounted clips: OVESCO Padlock system Endoscopic band ligation	Contact: Heater probe Bipolar Monopolar Noncontact: Argon plasma coagulation	Hemospray (TC-325) Ankaferd blood stopper Endoclot
